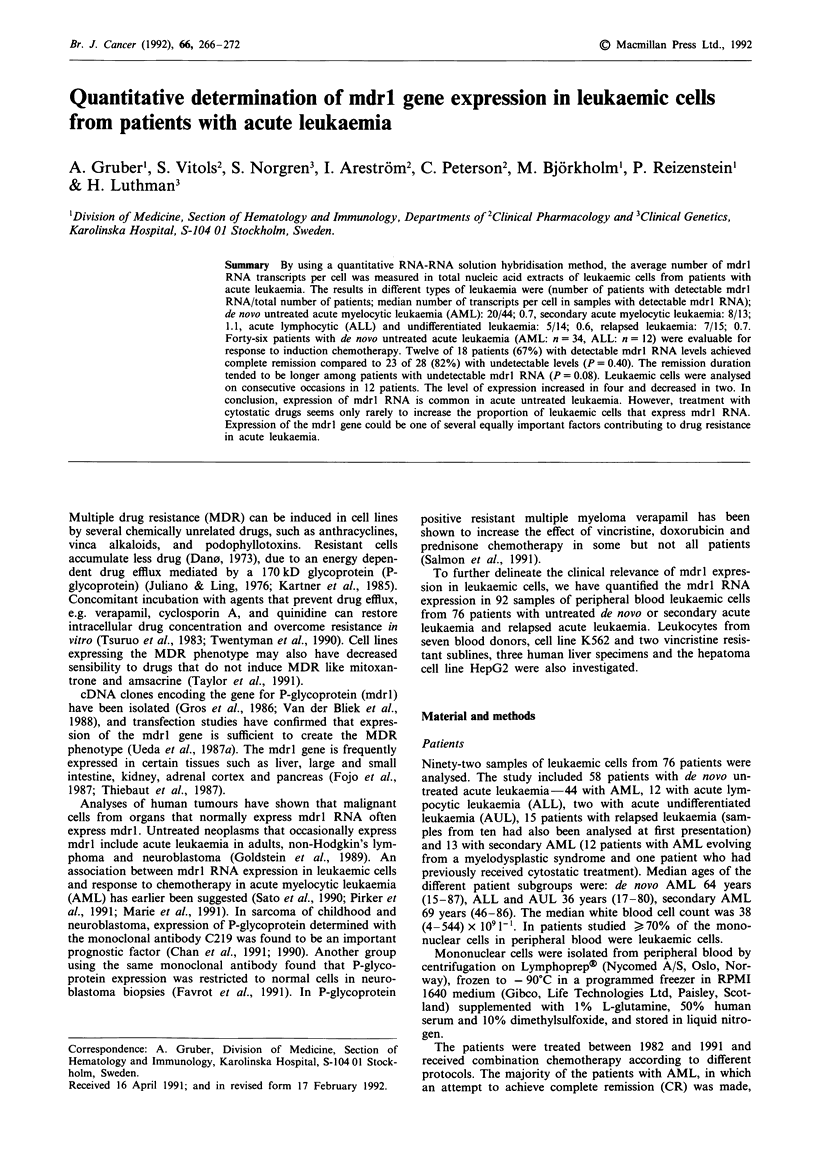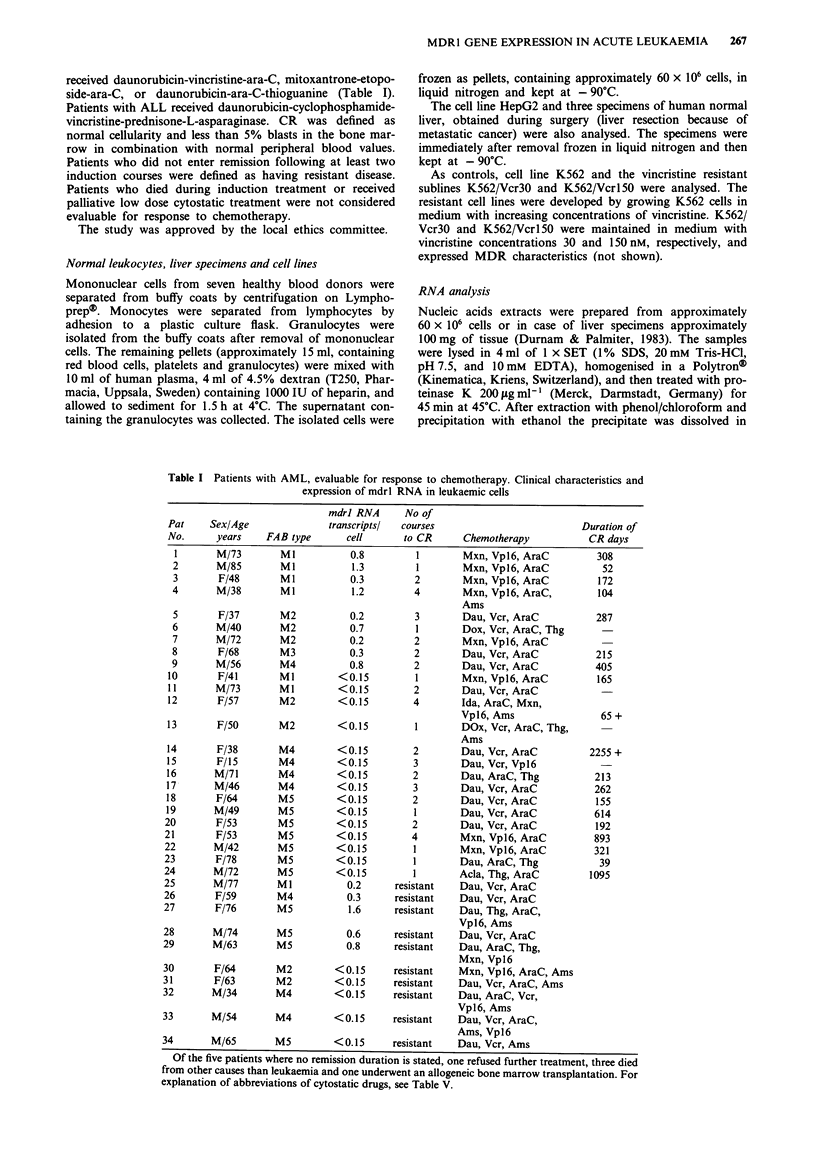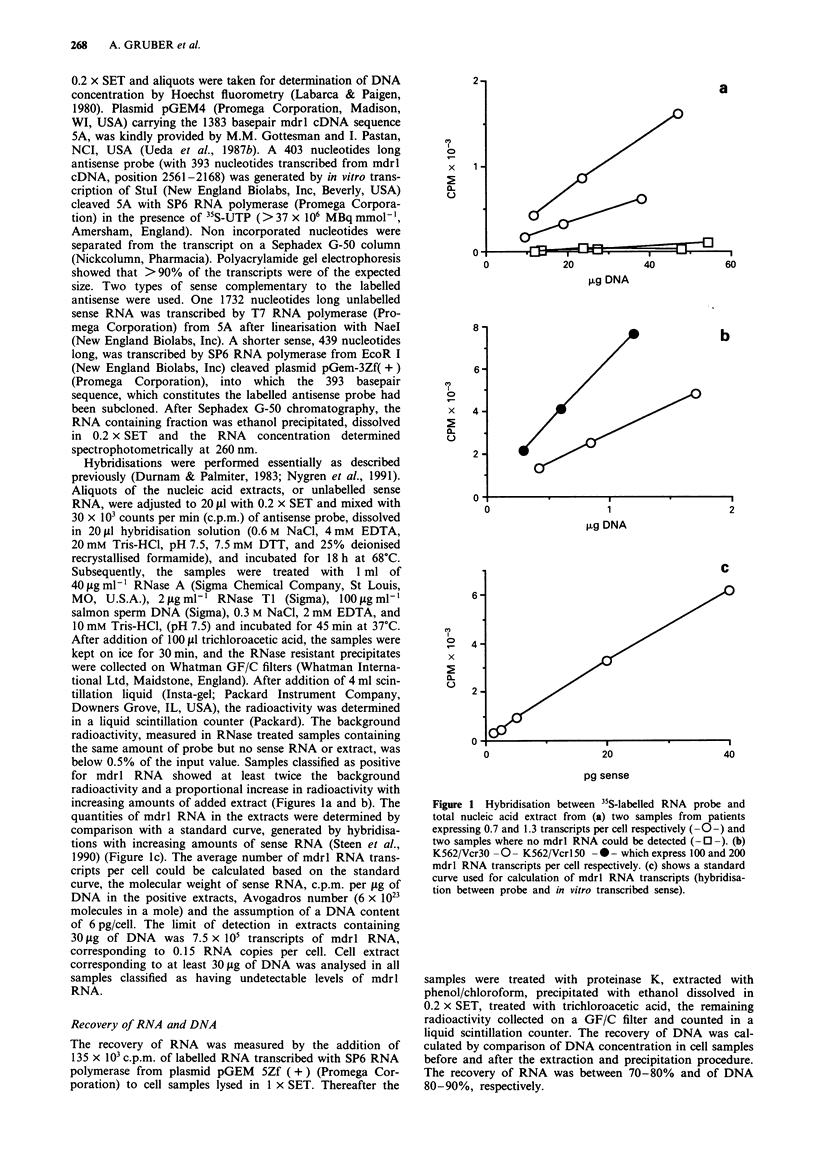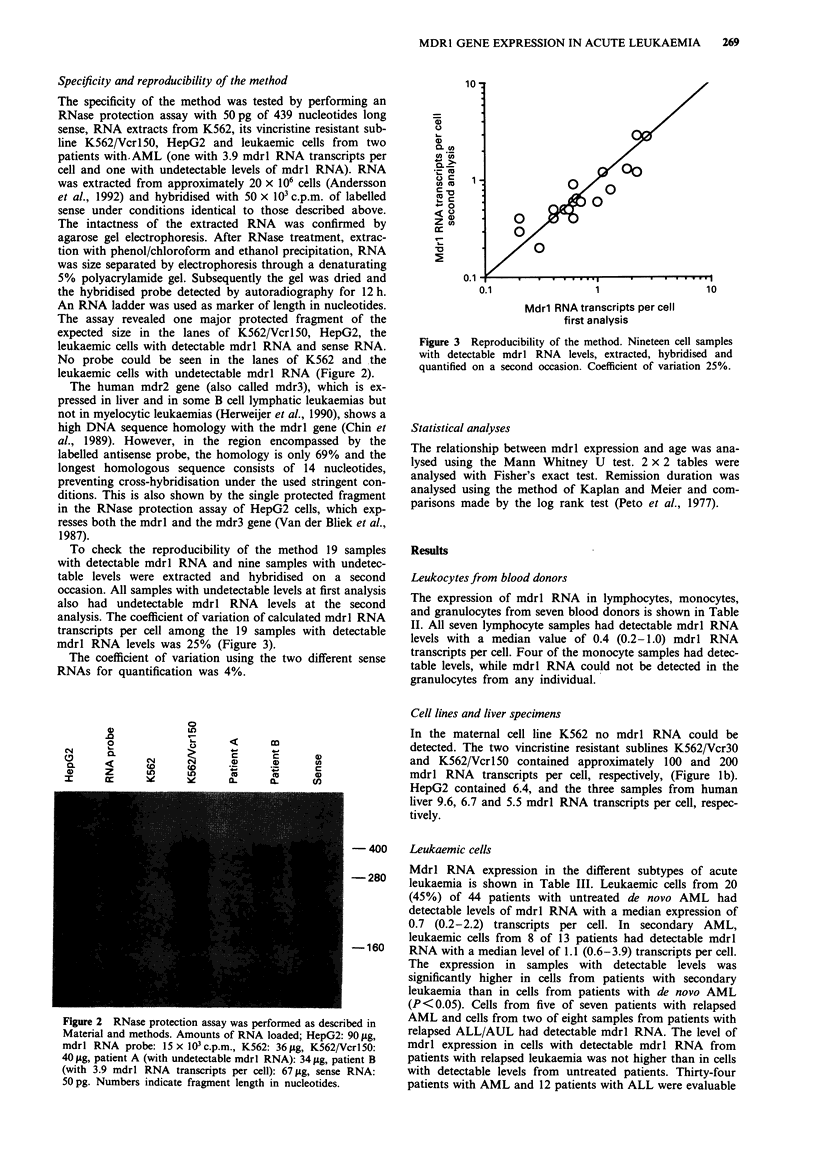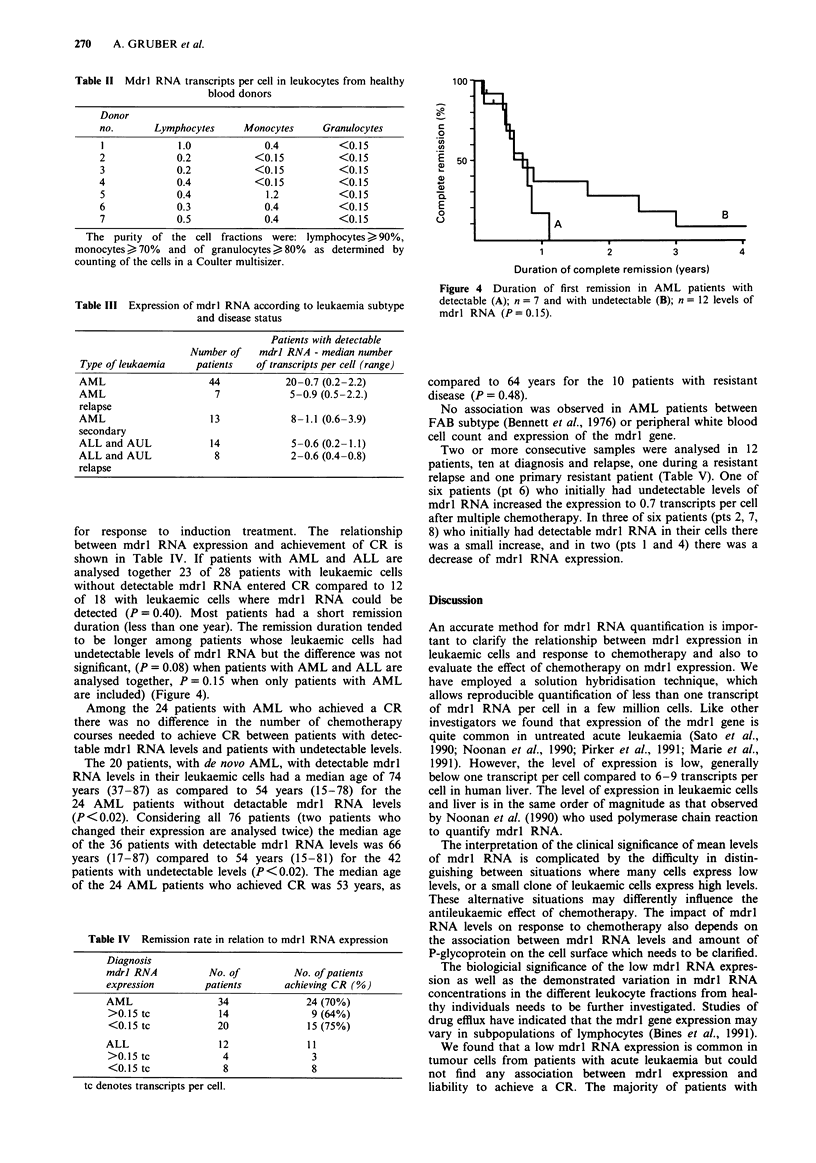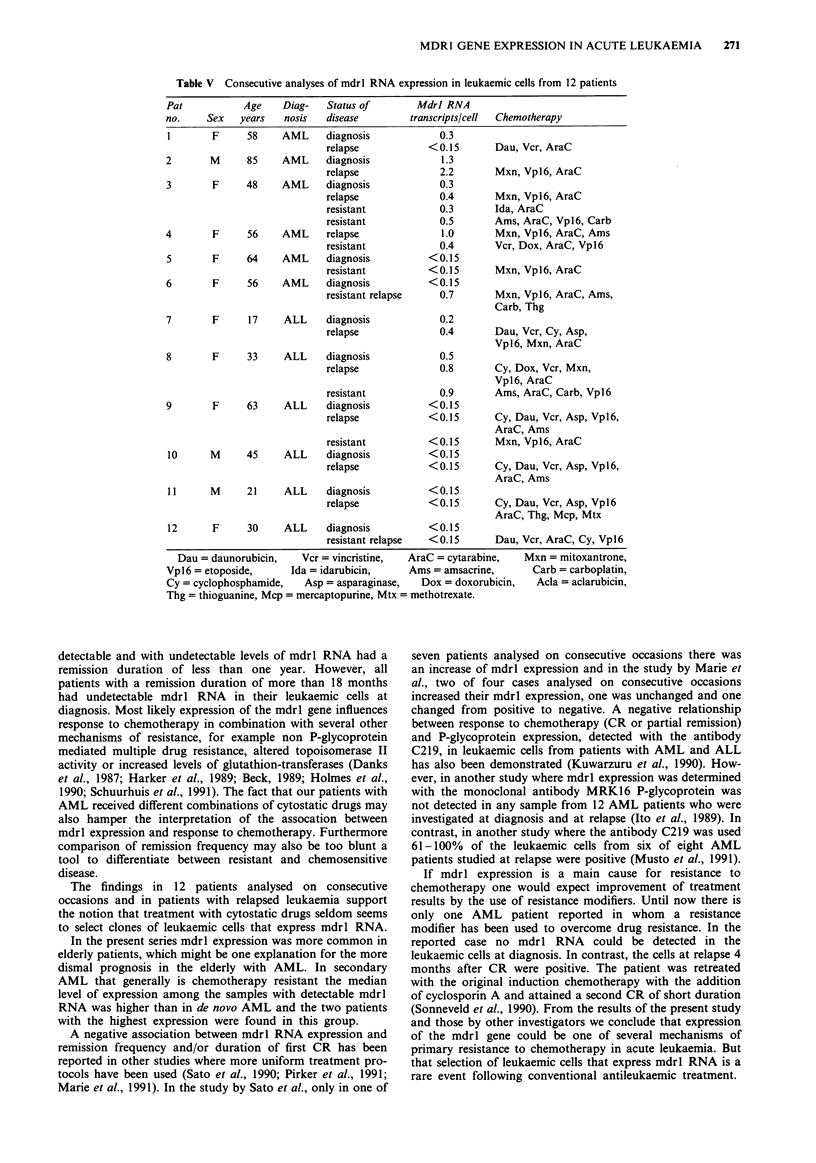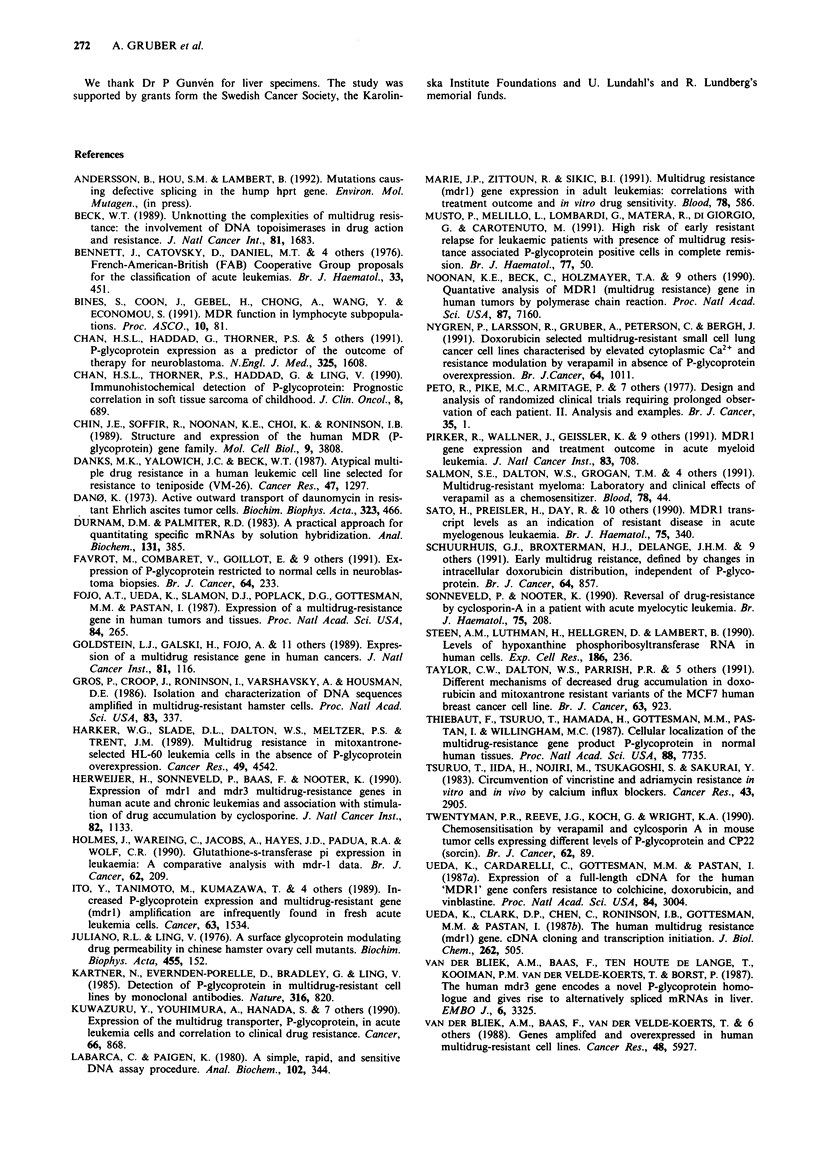# Quantitative determination of mdr1 gene expression in leukaemic cells from patients with acute leukaemia.

**DOI:** 10.1038/bjc.1992.255

**Published:** 1992-08

**Authors:** A. Gruber, S. Vitols, S. Norgren, I. Areström, C. Peterson, M. Björkholm, P. Reizenstein, H. Luthman

**Affiliations:** Division of Medicine, Karolinska Hospital, Stockholm, Sweden.

## Abstract

**Images:**


Br. J. Cancer (1992), 66, 266-272 © Macmillan Press Ltd., 1992

### Quantitative determination of mdr1 gene expression in leukaemic cells from patients with acute leukaemia

A. Gruber[1], S. Vitols[2], S. Norgren[3], I. Areström[2], C. Peterson[2], M. Björkholm[1], P. Reizenstein[1] & H. Luthman[3]

[1]*Division of Medicine, Section of Hematology and Immunology, Departments of* [2]*Clinical Pharmacology and* [3]*Clinical Genetics, Karolinska Hospital, S-104 01 Stockholm, Sweden.*

**Summary** By using a quantitative RNA-RNA solution hybridisation method, the average number of mdr1 RNA transcripts per cell was measured in total nucleic acid extracts of leukaemic cells from patients with acute leukaemia. The results in different types of leukaemia were (number of patients with detectable mdr1 RNA/total number of patients; median number of transcripts per cell in samples with detectable mdr1 RNA); *de novo* untreated acute myelocytic leukaemia (AML): 20/44; 0.7, secondary acute myelocytic leukaemia: 8/13; 1.1, acute lymphocytic (ALL) and undifferentiated leukaemia: 5/14; 0.6, relapsed leukaemia: 7/15; 0.7. Forty-six patients with *de novo* untreated acute leukaemia (AML: $n = 34$, ALL: $n = 12$) were evaluable for response to induction chemotherapy. Twelve of 18 patients (67%) with detectable mdr1 RNA levels achieved complete remission compared to 23 of 28 (82%) with undetectable levels ($P = 0.40$). The remission duration tended to be longer among patients with undetectable mdr1 RNA ($P = 0.08$). Leukaemic cells were analysed on consecutive occasions in 12 patients. The level of expression increased in four and decreased in two. In conclusion, expression of mdr1 RNA is common in acute untreated leukaemia. However, treatment with cytostatic drugs seems only rarely to increase the proportion of leukaemic cells that express mdr1 RNA. Expression of the mdr1 gene could be one of several equally important factors contributing to drug resistance in acute leukaemia.

Multiple drug resistance (MDR) can be induced in cell lines by several chemically unrelated drugs, such as anthracyclines, vinca alkaloids, and podophyllotoxins. Resistant cells accumulate less drug (Danø, 1973), due to an energy dependent drug efflux mediated by a 170 kD glycoprotein (P-glycoprotein) (Juliano & Ling, 1976; Kartner *et al.*, 1985). Concomitant incubation with agents that prevent drug efflux, e.g. verapamil, cyclosporin A, and quinidine can restore intracellular drug concentration and overcome resistance *in vitro* (Tsuruo *et al.*, 1983; Twentyman *et al.*, 1990). Cell lines expressing the MDR phenotype may also have decreased sensibility to drugs that do not induce MDR like mitoxantrone and amsacrine (Taylor *et al.*, 1991).

cDNA clones encoding the gene for P-glycoprotein (mdr1) have been isolated (Gros *et al.*, 1986; Van der Bliek *et al.*, 1988), and transfection studies have confirmed that expression of the mdr1 gene is sufficient to create the MDR phenotype (Ueda *et al.*, 1987*a*). The mdr1 gene is frequently expressed in certain tissues such as liver, large and small intestine, kidney, adrenal cortex and pancreas (Fojo *et al.*, 1987; Thiebaut *et al.*, 1987).

Analyses of human tumours have shown that malignant cells from organs that normally express mdr1 RNA often express mdr1. Untreated neoplasms that occasionally express mdr1 include acute leukaemia in adults, non-Hodgkin's lymphoma and neuroblastoma (Goldstein *et al.*, 1989). An association between mdr1 RNA expression in leukaemic cells and response to chemotherapy in acute myelocytic leukaemia (AML) has earlier been suggested (Sato *et al.*, 1990; Pirker *et al.*, 1991; Marie *et al.*, 1991). In sarcoma of childhood and neuroblastoma, expression of P-glycoprotein determined with the monoclonal antibody C219 was found to be an important prognostic factor (Chan *et al.*, 1991; 1990). Another group using the same monoclonal antibody found that P-glycoprotein expression was restricted to normal cells in neuroblastoma biopsies (Favrot *et al.*, 1991). In P-glycoprotein positive resistant multiple myeloma verapamil has been shown to increase the effect of vincristine, doxorubicin and prednisone chemotherapy in some but not all patients (Salmon *et al.*, 1991).

To further delineate the clinical relevance of mdr1 expression in leukaemic cells, we have quantified the mdr1 RNA expression in 92 samples of peripheral blood leukaemic cells from 76 patients with untreated *de novo* or secondary acute leukaemia and relapsed acute leukaemia. Leukocytes from seven blood donors, cell line K562 and two vincristine resistant sublines, three human liver specimens and the hepatoma cell line HepG2 were also investigated.

### Material and methods

### *Patients*

Ninety-two samples of leukaemic cells from 76 patients were analysed. The study included 58 patients with *de novo* untreated acute leukaemia—44 with AML, 12 with acute lympocytic leukaemia (ALL), two with acute undifferentiated leukaemia (AUL), 15 patients with relapsed leukaemia (samples from ten had also been analysed at first presentation) and 13 with secondary AML (12 patients with AML evolving from a myelodysplastic syndrome and one patient who had previously received cytostatic treatment). Median ages of the different patient subgroups were: *de novo* AML 64 years (15-87), ALL and AUL 36 years (17-80), secondary AML 69 years (46-86). The median white blood cell count was 38 (4-544) $\times 10^9 l^{-1}$. In patients studied $\geqslant$70% of the mononuclear cells in peripheral blood were leukaemic cells.

Mononuclear cells were isolated from peripheral blood by centrifugation on Lymphoprep® (Nycomed A/S, Oslo, Norway), frozen to $-90°C$ in a programmed freezer in RPMI 1640 medium (Gibco, Life Technologies Ltd, Paisley, Scotland) supplemented with 1% L-glutamine, 50% human serum and 10% dimethylsulfoxide, and stored in liquid nitrogen.

The patients were treated between 1982 and 1991 and received combination chemotherapy according to different protocols. The majority of the patients with AML, in which an attempt to achieve complete remission (CR) was made,

Correspondence: A. Gruber, Division of Medicine, Section of Hematology and Immunology, Karolinska Hospital, S-104 01 Stockholm, Sweden.
Received 16 April 1991; and in revised form 17 February 1992.

received daunorubicin-vincristine-ara-C, mitoxantrone-etoposide-ara-C, or daunorubicin-ara-C-thioguanine (Table I). Patients with ALL received daunorubicin-cyclophosphamide-vincristine-prednisone-L-asparaginase. CR was defined as normal cellularity and less than 5% blasts in the bone marrow in combination with normal peripheral blood values. Patients who did not enter remission following at least two induction courses were defined as having resistant disease. Patients who died during induction treatment or received palliative low dose cytostatic treatment were not considered evaluable for response to chemotherapy.

The study was approved by the local ethics committee.

### *Normal leukocytes, liver specimens and cell lines*

Mononuclear cells from seven healthy blood donors were separated from buffy coats by centrifugation on Lymphoprep®. Monocytes were separated from lymphocytes by adhesion to a plastic culture flask. Granulocytes were isolated from the buffy coats after removal of mononuclear cells. The remaining pellets (approximately 15 ml, containing red blood cells, platelets and granulocytes) were mixed with 10 ml of human plasma, 4 ml of 4.5% dextran (T250, Pharmacia, Uppsala, Sweden) containing 1000 IU of heparin, and allowed to sediment for 1.5 h at 4°C. The supernatant containing the granulocytes was collected. The isolated cells were frozen as pellets, containing approximately $60 \times 10^6$ cells, in liquid nitrogen and kept at − 90°C.

The cell line HepG2 and three specimens of human normal liver, obtained during surgery (liver resection because of metastatic cancer) were also analysed. The specimens were immediately after removal frozen in liquid nitrogen and then kept at − 90°C.

As controls, cell line K562 and the vincristine resistant sublines K562/Vcr30 and K562/Vcr150 were analysed. The resistant cell lines were developed by growing K562 cells in medium with increasing concentrations of vincristine. K562/Vcr30 and K562/Vcr150 were maintained in medium with vincristine concentrations 30 and 150 nM, respectively, and expressed MDR characteristics (not shown).

### *RNA analysis*

Nucleic acids extracts were prepared from approximately $60 \times 10^6$ cells or in case of liver specimens approximately 100 mg of tissue (Durnam & Palmiter, 1983). The samples were lysed in 4 ml of 1 × SET (1% SDS, 20 mM Tris-HCl, pH 7.5, and 10 mM EDTA), homogenised in a Polytron® (Kinematica, Kriens, Switzerland), and then treated with proteinase K 200 $\mu g\ ml^{-1}$ (Merck, Darmstadt, Germany) for 45 min at 45°C. After extraction with phenol/chloroform and precipitation with ethanol the precipitate was dissolved in

**Table I** Patients with AML, evaluable for response to chemotherapy. Clinical characteristics and expression of mdr1 RNA in leukaemic cells

| *Pat No.* | *Sex/Age years* | *FAB type* | *mdr1 RNA transcripts/ cell* | *No of courses to CR* | *Chemotherapy* | *Duration of CR days* |
|---|---|---|---|---|---|---|
| 1 | M/73 | M1 | 0.8 | 1 | Mxn, Vp16, AraC | 308 |
| 2 | M/85 | M1 | 1.3 | 1 | Mxn, Vp16, AraC | 52 |
| 3 | F/48 | M1 | 0.3 | 2 | Mxn, Vp16, AraC | 172 |
| 4 | M/38 | M1 | 1.2 | 4 | Mxn, Vp16, AraC, Ams | 104 |
| 5 | F/37 | M2 | 0.2 | 3 | Dau, Vcr, AraC | 287 |
| 6 | M/40 | M2 | 0.7 | 1 | Dox, Vcr, AraC, Thg | — |
| 7 | M/72 | M2 | 0.2 | 2 | Mxn, Vp16, AraC | — |
| 8 | F/68 | M3 | 0.3 | 2 | Dau, Vcr, AraC | 215 |
| 9 | M/56 | M4 | 0.8 | 2 | Dau, Vcr, AraC | 405 |
| 10 | F/41 | M1 | <0.15 | 1 | Mxn, Vp16, AraC | 165 |
| 11 | M/73 | M1 | <0.15 | 2 | Dau, Vcr, AraC | — |
| 12 | F/57 | M2 | <0.15 | 4 | Ida, AraC, Mxn, Vp16, Ams | 65 + |
| 13 | F/50 | M2 | <0.15 | 1 | DOx, Vcr, AraC, Thg, Ams | — |
| 14 | F/38 | M4 | <0.15 | 2 | Dau, Vcr, AraC | 2255 + |
| 15 | F/15 | M4 | <0.15 | 3 | Dau, Vcr, Vp16 | — |
| 16 | M/71 | M4 | <0.15 | 2 | Dau, AraC, Thg | 213 |
| 17 | M/46 | M4 | <0.15 | 3 | Dau, Vcr, AraC | 262 |
| 18 | F/64 | M5 | <0.15 | 2 | Dau, Vcr, AraC | 155 |
| 19 | M/49 | M5 | <0.15 | 1 | Dau, Vcr, AraC | 614 |
| 20 | F/53 | M5 | <0.15 | 2 | Dau, Vcr, AraC | 192 |
| 21 | F/53 | M5 | <0.15 | 4 | Mxn, Vp16, AraC | 893 |
| 22 | M/42 | M5 | <0.15 | 1 | Mxn, Vp16, AraC | 321 |
| 23 | F/78 | M5 | <0.15 | 1 | Dau, AraC, Thg | 39 |
| 24 | M/72 | M5 | <0.15 | 1 | Acla, Thg, AraC | 1095 |
| 25 | M/77 | M1 | 0.2 | resistant | Dau, Vcr, AraC | |
| 26 | F/59 | M4 | 0.3 | resistant | Dau, Vcr, AraC | |
| 27 | F/76 | M5 | 1.6 | resistant | Dau, Thg, AraC, Vp16, Ams | |
| 28 | M/74 | M5 | 0.6 | resistant | Dau, Vcr, AraC | |
| 29 | M/63 | M5 | 0.8 | resistant | Dau, AraC, Thg, Mxn, Vp16 | |
| 30 | F/64 | M2 | <0.15 | resistant | Mxn, Vp16, AraC, Ams | |
| 31 | F/63 | M2 | <0.15 | resistant | Dau, Vcr, AraC, Ams | |
| 32 | M/34 | M4 | <0.15 | resistant | Dau, AraC, Vcr, Vp16, Ams | |
| 33 | M/54 | M4 | <0.15 | resistant | Dau, Vcr, AraC, Ams, Vp16 | |
| 34 | M/65 | M5 | <0.15 | resistant | Dau, Vcr, Ams | |

Of the five patients where no remission duration is stated, one refused further treatment, three died from other causes than leukaemia and one underwent an allogeneic bone marrow transplantation. For explanation of abbreviations of cytostatic drugs, see Table V.

0.2 × SET and aliquots were taken for determination of DNA concentration by Hoechst fluorometry (Labarca & Paigen, 1980). Plasmid pGEM4 (Promega Corporation, Madison, WI, USA) carrying the 1383 basepair mdr1 cDNA sequence 5A, was kindly provided by M.M. Gottesman and I. Pastan, NCI, USA (Ueda *et al.*, 1987*b*). A 403 nucleotides long antisense probe (with 393 nucleotides transcribed from mdr1 cDNA, position 2561–2168) was generated by *in vitro* transcription of StuI (New England Biolabs, Inc, Beverly, USA) cleaved 5A with SP6 RNA polymerase (Promega Corporation) in the presence of $^{35}$S-UTP ($>37 \times 10^6$ MBq mmol$^{-1}$, Amersham, England). Non incorporated nucleotides were separated from the transcript on a Sephadex G-50 column (Nickcolumn, Pharmacia). Polyacrylamide gel electrophoresis showed that >90% of the transcripts were of the expected size. Two types of sense complementary to the labelled antisense were used. One 1732 nucleotides long unlabelled sense RNA was transcribed by T7 RNA polymerase (Promega Corporation) from 5A after linearisation with NaeI (New England Biolabs, Inc). A shorter sense, 439 nucleotides long, was transcribed by SP6 RNA polymerase from EcoR I (New England Biolabs, Inc) cleaved plasmid pGem-3Zf( + ) (Promega Corporation), into which the 393 basepair sequence, which constitutes the labelled antisense probe had been subcloned. After Sephadex G-50 chromatography, the RNA containing fraction was ethanol precipitated, dissolved in 0.2 × SET and the RNA concentration determined spectrophotometrically at 260 nm.

Hybridisations were performed essentially as described previously (Durnam & Palmiter, 1983; Nygren *et al.*, 1991). Aliquots of the nucleic acid extracts, or unlabelled sense RNA, were adjusted to 20 µl with 0.2 × SET and mixed with $30 \times 10^3$ counts per min (c.p.m.) of antisense probe, dissolved in 20 µl hybridisation solution (0.6 M NaCl, 4 mM EDTA, 20 mM Tris-HCl, pH 7.5, 7.5 mM DTT, and 25% deionised recrystallised formamide), and incubated for 18 h at 68°C. Subsequently, the samples were treated with 1 ml of 40 µg ml$^{-1}$ RNase A (Sigma Chemical Company, St Louis, MO, U.S.A.), 2 µg ml$^{-1}$ RNase T1 (Sigma), 100 µg ml$^{-1}$ salmon sperm DNA (Sigma), 0.3 M NaCl, 2 mM EDTA, and 10 mM Tris-HCl, (pH 7.5) and incubated for 45 min at 37°C. After addition of 100 µl trichloroacetic acid, the samples were kept on ice for 30 min, and the RNase resistant precipitates were collected on Whatman GF/C filters (Whatman International Ltd, Maidstone, England). After addition of 4 ml scintillation liquid (Insta-gel; Packard Instrument Company, Downers Grove, IL, USA), the radioactivity was determined in a liquid scintillation counter (Packard). The background radioactivity, measured in RNase treated samples containing the same amount of probe but no sense RNA or extract, was below 0.5% of the input value. Samples classified as positive for mdr1 RNA showed at least twice the background radioactivity and a proportional increase in radioactivity with increasing amounts of added extract (Figures 1a and b). The quantities of mdr1 RNA in the extracts were determined by comparison with a standard curve, generated by hybridisations with increasing amounts of sense RNA (Steen *et al.*, 1990) (Figure 1c). The average number of mdr1 RNA transcripts per cell could be calculated based on the standard curve, the molecular weight of sense RNA, c.p.m. per µg of DNA in the positive extracts, Avogadros number ($6 \times 10^{23}$ molecules in a mole) and the assumption of a DNA content of 6 pg/cell. The limit of detection in extracts containing 30 µg of DNA was $7.5 \times 10^5$ transcripts of mdr1 RNA, corresponding to 0.15 RNA copies per cell. Cell extract corresponding to at least 30 µg of DNA was analysed in all samples classified as having undetectable levels of mdr1 RNA.

*Recovery of RNA and DNA*

The recovery of RNA was measured by the addition of $135 \times 10^3$ c.p.m. of labelled RNA transcribed with SP6 RNA polymerase from plasmid pGEM 5Zf ( + ) (Promega Corporation) to cell samples lysed in 1 × SET. Thereafter the samples were treated with proteinase K, extracted with phenol/chloroform, precipitated with ethanol dissolved in 0.2 × SET, treated with trichloroacetic acid, the remaining radioactivity collected on a GF/C filter and counted in a liquid scintillation counter. The recovery of DNA was calculated by comparison of DNA concentration in cell samples before and after the extraction and precipitation procedure. The recovery of RNA was between 70–80% and of DNA 80–90%, respectively.

**Figure 1** Hybridisation between $^{35}$S-labelled RNA probe and total nucleic acid extract from (**a**) two samples from patients expressing 0.7 and 1.3 transcripts per cell respectively (–○–) and two samples where no mdr1 RNA could be detected (–□–). (**b**) K562/Vcr30 –○– K562/Vcr150 –●– which express 100 and 200 mdr1 RNA transcripts per cell respectively. (**c**) shows a standard curve used for calculation of mdr1 RNA transcripts (hybridisation between probe and *in vitro* transcribed sense).

*Specificity and reproducibility of the method*

The specificity of the method was tested by performing an RNase protection assay with 50 pg of 439 nucleotides long sense, RNA extracts from K562, its vincristine resistant subline K562/Vcr150, HepG2 and leukaemic cells from two patients with AML (one with 3.9 mdr1 RNA transcripts per cell and one with undetectable levels of mdr1 RNA). RNA was extracted from approximately $20 \times 10^6$ cells (Andersson *et al.*, 1992) and hybridised with $50 \times 10^3$ c.p.m. of labelled sense under conditions identical to those described above. The intactness of the extracted RNA was confirmed by agarose gel electrophoresis. After RNase treatment, extraction with phenol/chloroform and ethanol precipitation, RNA was size separated by electrophoresis through a denaturating 5% polyacrylamide gel. Subsequently the gel was dried and the hybridised probe detected by autoradiography for 12 h. An RNA ladder was used as marker of length in nucleotides. The assay revealed one major protected fragment of the expected size in the lanes of K562/Vcr150, HepG2, the leukaemic cells with detectable mdr1 RNA and sense RNA. No probe could be seen in the lanes of K562 and the leukaemic cells with undetectable mdr1 RNA (Figure 2).

The human mdr2 gene (also called mdr3), which is expressed in liver and in some B cell lymphatic leukaemias but not in myelocytic leukaemias (Herweijer *et al.*, 1990), shows a high DNA sequence homology with the mdr1 gene (Chin *et al.*, 1989). However, in the region encompassed by the labelled antisense probe, the homology is only 69% and the longest homologous sequence consists of 14 nucleotides, preventing cross-hybridisation under the used stringent conditions. This is also shown by the single protected fragment in the RNase protection assay of HepG2 cells, which expresses both the mdr1 and the mdr3 gene (Van der Bliek *et al.*, 1987).

To check the reproducibility of the method 19 samples with detectable mdr1 RNA and nine samples with undetectable levels were extracted and hybridised on a second occasion. All samples with undetectable levels at first analysis also had undetectable mdr1 RNA levels at the second analysis. The coefficient of variation of calculated mdr1 RNA transcripts per cell among the 19 samples with detectable mdr1 RNA levels was 25% (Figure 3).

The coefficient of variation using the two different sense RNAs for quantification was 4%.

**Figure 2** RNase protection assay was performed as described in Material and methods. Amounts of RNA loaded; HepG2: 90 µg, mdr1 RNA probe: $15 \times 10^3$ c.p.m., K562: 36 µg, K562/Vcr150: 40 µg, patient A (with undetectable mdr1 RNA): 34 µg, patient B (with 3.9 mdr1 RNA transcripts per cell): 67 µg, sense RNA: 50 pg. Numbers indicate fragment length in nucleotides.

**Figure 3** Reproducibility of the method. Nineteen cell samples with detectable mdr1 RNA levels, extracted, hybridised and quantified on a second occasion. Coefficient of variation 25%.

*Statistical analyses*

The relationship between mdr1 expression and age was analysed using the Mann Whitney U test. $2 \times 2$ tables were analysed with Fisher's exact test. Remission duration was analysed using the method of Kaplan and Meier and comparisons made by the log rank test (Peto *et al.*, 1977).

### Results

*Leukocytes from blood donors*

The expression of mdr1 RNA in lymphocytes, monocytes, and granulocytes from seven blood donors is shown in Table II. All seven lymphocyte samples had detectable mdr1 RNA levels with a median value of 0.4 (0.2–1.0) mdr1 RNA transcripts per cell. Four of the monocyte samples had detectable levels, while mdr1 RNA could not be detected in the granulocytes from any individual.

*Cell lines and liver specimens*

In the maternal cell line K562 no mdr1 RNA could be detected. The two vincristine resistant sublines K562/Vcr30 and K562/Vcr150 contained approximately 100 and 200 mdr1 RNA transcripts per cell, respectively, (Figure 1b). HepG2 contained 6.4, and the three samples from human liver 9.6, 6.7 and 5.5 mdr1 RNA transcripts per cell, respectively.

*Leukaemic cells*

Mdr1 RNA expression in the different subtypes of acute leukaemia is shown in Table III. Leukaemic cells from 20 (45%) of 44 patients with untreated *de novo* AML had detectable levels of mdr1 RNA with a median expression of 0.7 (0.2–2.2) transcripts per cell. In secondary AML, leukaemic cells from 8 of 13 patients had detectable mdr1 RNA with a median level of 1.1 (0.6–3.9) transcripts per cell. The expression in samples with detectable levels was significantly higher in cells from patients with secondary leukaemia than in cells from patients with *de novo* AML ($P < 0.05$). Cells from five of seven patients with relapsed AML and cells from two of eight samples from patients with relapsed ALL/AUL had detectable mdr1 RNA. The level of mdr1 expression in cells with detectable mdr1 RNA from patients with relapsed leukaemia was not higher than in cells with detectable levels from untreated patients. Thirty-four patients with AML and 12 patients with ALL were evaluable

Table II Mdr1 RNA transcripts per cell in leukocytes from healthy blood donors

| Donor no. | Lymphocytes | Monocytes | Granulocytes |
|---|---|---|---|
| 1 | 1.0 | 0.4 | <0.15 |
| 2 | 0.2 | <0.15 | <0.15 |
| 3 | 0.2 | <0.15 | <0.15 |
| 4 | 0.4 | <0.15 | <0.15 |
| 5 | 0.4 | 1.2 | <0.15 |
| 6 | 0.3 | 0.4 | <0.15 |
| 7 | 0.5 | 0.4 | <0.15 |

The purity of the cell fractions were: lymphocytes ⩾ 90%, monocytes ⩾ 70% and of granulocytes ⩾ 80% as determined by counting of the cells in a Coulter multisizer.

Table III Expression of mdr1 RNA according to leukaemia subtype and disease status

| Type of leukaemia | Number of patients | Patients with detectable mdr1 RNA - median number of transcripts per cell (range) |
|---|---|---|
| AML | 44 | 20-0.7 (0.2-2.2) |
| AML relapse | 7 | 5-0.9 (0.5-2.2.) |
| AML secondary | 13 | 8-1.1 (0.6-3.9) |
| ALL and AUL | 14 | 5-0.6 (0.2-1.1) |
| ALL and AUL relapse | 8 | 2-0.6 (0.4-0.8) |

for response to induction treatment. The relationship between mdr1 RNA expression and achievement of CR is shown in Table IV. If patients with AML and ALL are analysed together 23 of 28 patients with leukaemic cells without detectable mdr1 RNA entered CR compared to 12 of 18 with leukaemic cells where mdr1 RNA could be detected ($P = 0.40$). Most patients had a short remission duration (less than one year). The remission duration tended to be longer among patients whose leukaemic cells had undetectable levels of mdr1 RNA but the difference was not significant, ($P = 0.08$) when patients with AML and ALL are analysed together, $P = 0.15$ when only patients with AML are included) (Figure 4).

Among the 24 patients with AML who achieved a CR there was no difference in the number of chemotherapy courses needed to achieve CR between patients with detectable mdr1 RNA levels and patients with undetectable levels.

The 20 patients, with *de novo* AML, with detectable mdr1 RNA levels in their leukaemic cells had a median age of 74 years (37–87) as compared to 54 years (15–78) for the 24 AML patients without detactable mdr1 RNA levels ($P < 0.02$). Considering all 76 patients (two patients who changed their expression are analysed twice) the median age of the 36 patients with detectable mdr1 RNA levels was 66 years (17–87) compared to 54 years (15–81) for the 42 patients with undetectable levels ($P < 0.02$). The median age of the 24 AML patients who achieved CR was 53 years, as

Table IV Remission rate in relation to mdr1 RNA expression

| Diagnosis mdr1 RNA expression | No. of patients | No. of patients achieving CR (%) |
|---|---|---|
| AML | 34 | 24 (70%) |
| >0.15 tc | 14 | 9 (64%) |
| <0.15 tc | 20 | 15 (75%) |
| ALL | 12 | 11 |
| >0.15 tc | 4 | 3 |
| <0.15 tc | 8 | 8 |

tc denotes transcripts per cell.

**Figure 4** Duration of first remission in AML patients with detectable (**A**); $n = 7$ and with undetectable (**B**); $n = 12$ levels of mdr1 RNA ($P = 0.15$).

compared to 64 years for the 10 patients with resistant disease ($P = 0.48$).

No association was observed in AML patients between FAB subtype (Bennett *et al.*, 1976) or peripheral white blood cell count and expression of the mdr1 gene.

Two or more consecutive samples were analysed in 12 patients, ten at diagnosis and relapse, one during a resistant relapse and one primary resistant patient (Table V). One of six patients (pt 6) who initially had undetectable levels of mdr1 RNA increased the expression to 0.7 transcripts per cell after multiple chemotherapy. In three of six patients (pts 2, 7, 8) who initially had detectable mdr1 RNA in their cells there was a small increase, and in two (pts 1 and 4) there was a decrease of mdr1 RNA expression.

### Discussion

An accurate method for mdr1 RNA quantification is important to clarify the relationship between mdr1 expression in leukaemic cells and response to chemotherapy and also to evaluate the effect of chemotherapy on mdr1 expression. We have employed a solution hybridisation technique, which allows reproducible quantification of less than one transcript of mdr1 RNA per cell in a few million cells. Like other investigators we found that expression of the mdr1 gene is quite common in untreated acute leukaemia (Sato *et al.*, 1990; Noonan *et al.*, 1990; Pirker *et al.*, 1991; Marie *et al.*, 1991). However, the level of expression is low, generally below one transcript per cell compared to 6–9 transcripts per cell in human liver. The level of expression in leukaemic cells and liver is in the same order of magnitude as that observed by Noonan *et al.* (1990) who used polymerase chain reaction to quantify mdr1 RNA.

The interpretation of the clinical significance of mean levels of mdr1 RNA is complicated by the difficulty in distinguishing between situations where many cells express low levels, or a small clone of leukaemic cells express high levels. These alternative situations may differently influence the antileukaemic effect of chemotherapy. The impact of mdr1 RNA levels on response to chemotherapy also depends on the association between mdr1 RNA levels and amount of P-glycoprotein on the cell surface which needs to be clarified.

The biologicial significance of the low mdr1 RNA expression as well as the demonstrated variation in mdr1 RNA concentrations in the different leukocyte fractions from healthy individuals needs to be further investigated. Studies of drug efflux have indicated that the mdr1 gene expression may vary in subpopulations of lymphocytes (Bines *et al.*, 1991).

We found that a low mdr1 RNA expression is common in tumour cells from patients with acute leukaemia but could not find any association between mdr1 expression and liability to achieve a CR. The majority of patients with

**Table V** Consecutive analyses of mdr1 RNA expression in leukaemic cells from 12 patients

| *Pat no.* | *Sex* | *Age years* | *Diag-nosis* | *Status of disease* | *Mdr1 RNA transcripts/cell* | *Chemotherapy* |
|---|---|---|---|---|---|---|
| 1 | F | 58 | AML | diagnosis | 0.3 | |
| | | | | relapse | <0.15 | Dau, Vcr, AraC |
| 2 | M | 85 | AML | diagnosis | 1.3 | |
| | | | | relapse | 2.2 | Mxn, Vp16, AraC |
| 3 | F | 48 | AML | diagnosis | 0.3 | |
| | | | | relapse | 0.4 | Mxn, Vp16, AraC |
| | | | | resistant | 0.3 | Ida, AraC |
| | | | | resistant | 0.5 | Ams, AraC, Vp16, Carb |
| 4 | F | 56 | AML | relapse | 1.0 | Mxn, Vp16, AraC, Ams |
| | | | | resistant | 0.4 | Vcr, Dox, AraC, Vp16 |
| 5 | F | 64 | AML | diagnosis | <0.15 | |
| | | | | resistant | <0.15 | Mxn, Vp16, AraC |
| 6 | F | 56 | AML | diagnosis | <0.15 | |
| | | | | resistant relapse | 0.7 | Mxn, Vp16, AraC, Ams, Carb, Thg |
| 7 | F | 17 | ALL | diagnosis | 0.2 | |
| | | | | relapse | 0.4 | Dau, Vcr, Cy, Asp, Vp16, Mxn, AraC |
| 8 | F | 33 | ALL | diagnosis | 0.5 | |
| | | | | relapse | 0.8 | Cy, Dox, Vcr, Mxn, Vp16, AraC |
| | | | | resistant | 0.9 | Ams, AraC, Carb, Vp16 |
| 9 | F | 63 | ALL | diagnosis | <0.15 | |
| | | | | relapse | <0.15 | Cy, Dau, Vcr, Asp, Vp16, AraC, Ams |
| | | | | resistant | <0.15 | Mxn, Vp16, AraC |
| 10 | M | 45 | ALL | diagnosis | <0.15 | |
| | | | | relapse | <0.15 | Cy, Dau, Vcr, Asp, Vp16, AraC, Ams |
| 11 | M | 21 | ALL | diagnosis | <0.15 | |
| | | | | relapse | <0.15 | Cy, Dau, Vcr, Asp, Vp16 AraC, Thg, Mcp, Mtx |
| 12 | F | 30 | ALL | diagnosis | <0.15 | |
| | | | | resistant relapse | <0.15 | Dau, Vcr, AraC, Cy, Vp16 |

Dau = daunorubicin, Vcr = vincristine, AraC = cytarabine, Mxn = mitoxantrone, Vp16 = etoposide, Ida = idarubicin, Ams = amsacrine, Carb = carboplatin, Cy = cyclophosphamide, Asp = asparaginase, Dox = doxorubicin, Acla = aclarubicin, Thg = thioguanine, Mcp = mercaptopurine, Mtx = methotrexate.

detectable and with undetectable levels of mdr1 RNA had a remission duration of less than one year. However, all patients with a remission duration of more than 18 months had undetectable mdr1 RNA in their leukaemic cells at diagnosis. Most likely expression of the mdr1 gene influences response to chemotherapy in combination with several other mechanisms of resistance, for example non P-glycoprotein mediated multiple drug resistance, altered topoisomerase II activity or increased levels of glutathion-transferases (Danks *et al.*, 1987; Harker *et al.*, 1989; Beck, 1989; Holmes *et al.*, 1990; Schuurhuis *et al.*, 1991). The fact that our patients with AML received different combinations of cytostatic drugs may also hamper the interpretation of the assocation between mdr1 expression and response to chemotherapy. Furthermore comparison of remission frequency may also be too blunt a tool to differentiate between resistant and chemosensitive disease.

The findings in 12 patients analysed on consecutive occasions and in patients with relapsed leukaemia support the notion that treatment with cytostatic drugs seldom seems to select clones of leukaemic cells that express mdr1 RNA.

In the present series mdr1 expression was more common in elderly patients, which might be one explanation for the more dismal prognosis in the elderly with AML. In secondary AML that generally is chemotherapy resistant the median level of expression among the samples with detectable mdr1 RNA was higher than in *de novo* AML and the two patients with the highest expression were found in this group.

A negative association between mdr1 RNA expression and remission frequency and/or duration of first CR has been reported in other studies where more uniform treatment protocols have been used (Sato *et al.*, 1990; Pirker *et al.*, 1991; Marie *et al.*, 1991). In the study by Sato *et al.*, only in one of seven patients analysed on consecutive occasions there was an increase of mdr1 expression and in the study by Marie *et al.*, two of four cases analysed on consecutive occasions increased their mdr1 expression, one was unchanged and one changed from positive to negative. A negative relationship between response to chemotherapy (CR or partial remission) and P-glycoprotein expression, detected with the antibody C219, in leukaemic cells from patients with AML and ALL has also been demonstrated (Kuwarzuru *et al.*, 1990). However, in another study where mdr1 expression was determined with the monoclonal antibody MRK16 P-glycoprotein was not detected in any sample from 12 AML patients who were investigated at diagnosis and at relapse (Ito *et al.*, 1989). In contrast, in another study where the antibody C219 was used 61–100% of the leukaemic cells from six of eight AML patients studied at relapse were positive (Musto *et al.*, 1991).

If mdr1 expression is a main cause for resistance to chemotherapy one would expect improvement of treatment results by the use of resistance modifiers. Until now there is only one AML patient reported in whom a resistance modifier has been used to overcome drug resistance. In the reported case no mdr1 RNA could be detected in the leukaemic cells at diagnosis. In contrast, the cells at relapse 4 months after CR were positive. The patient was retreated with the original induction chemotherapy with the addition of cyclosporin A and attained a second CR of short duration (Sonneveld *et al.*, 1990). From the results of the present study and those by other investigators we conclude that expression of the mdr1 gene could be one of several mechanisms of primary resistance to chemotherapy in acute leukaemia. But that selection of leukaemic cells that express mdr1 RNA is a rare event following conventional antileukaemic treatment.

We thank Dr P Gunvén for liver specimens. The study was supported by grants form the Swedish Cancer Society, the Karolinska Institute Foundations and U. Lundahl's and R. Lundberg's memorial funds.

### References

ANDERSSON, B., HOU, S.M. & LAMBERT, B. (1992). Mutations causing defective splicing in the hump hprt gene. *Environ. Mol. Mutagen.*, (in press).

BECK, W.T. (1989). Unknotting the complexities of multidrug resistance: the involvement of DNA topoisimerases in drug action and resistance. *J. Natl Cancer Int.*, **81**, 1683.

BENNETT, J., CATOVSKY, D., DANIEL, M.T. & 4 others (1976). French-American-British (FAB) Cooperative Group proposals for the classification of acute leukemias. *Br. J. Haematol.*, **33**, 451.

BINES, S., COON, J., GEBEL, H., CHONG, A., WANG, Y. & ECONOMOU, S. (1991). MDR function in lymphocyte subpopulations. *Proc. ASCO.*, **10**, 81.

CHAN, H.S.L., HADDAD, G., THORNER, P.S. & 5 others (1991). P-glycoprotein expression as a predictor of the outcome of therapy for neuroblastoma. *N.Engl. J. Med.*, **325**, 1608.

CHAN, H.S.L., THORNER, P.S., HADDAD, G. & LING, V. (1990). Immunohistochemical detection of P-glycoprotein: Prognostic correlation in soft tissue sarcoma of childhood. *J. Clin. Oncol.*, **8**, 689.

CHIN, J.E., SOFFIR, R., NOONAN, K.E., CHOI, K. & RONINSON, I.B. (1989). Structure and expression of the human MDR (P-glycoprotein) gene family. *Mol. Cell Biol.*, **9**, 3808.

DANKS, M.K., YALOWICH, J.C. & BECK, W.T. (1987). Atypical multiple drug resistance in a human leukemic cell line selected for resistance to teniposide (VM-26). *Cancer Res.*, **47**, 1297.

DANØ, K. (1973). Active outward transport of daunomycin in resistant Ehrlich ascites tumor cells. *Biochim. Biophys. Acta.*, **323**, 466.

DURNAM, D.M. & PALMITER, R.D. (1983). A practical approach for quantitating specific mRNAs by solution hybridization. *Anal. Biochem.*, **131**, 385.

FAVROT, M., COMBARET, V., GOILLOT, E. & 9 others (1991). Expression of P-glycoprotein restricted to normal cells in neuroblastoma biopsies. *Br. J. Cancer*, **64**, 233.

FOJO, A.T., UEDA, K., SLAMON, D.J., POPLACK, D.G., GOTTESMAN, M.M. & PASTAN, I. (1987). Expression of a multidrug-resistance gene in human tumors and tissues. *Proc. Natl Acad. Sci. USA*, **84**, 265.

GOLDSTEIN, L.J., GALSKI, H., FOJO, A. & 11 others (1989). Expression of a multidrug resistance gene in human cancers. *J. Natl Cancer Inst.*, **81**, 116.

GROS, P., CROOP, J., RONINSON, I., VARSHAVSKY, A. & HOUSMAN, D.E. (1986). Isolation and characterization of DNA sequences amplified in multidrug-resistant hamster cells. *Proc. Natl Acad. Sci. USA*, **83**, 337.

HARKER, W.G., SLADE, D.L., DALTON, W.S., MELTZER, P.S. & TRENT, J.M. (1989). Multidrug resistance in mitoxantrone-selected HL-60 leukemia cells in the absence of P-glycoprotein overexpression. *Cancer Res.*, **49**, 4542.

HERWEIJER, H., SONNEVELD, P., BAAS, F. & NOOTER, K. (1990). Expression of mdr1 and mdr3 multidrug-resistance genes in human acute and chronic leukemias and association with stimulation of drug accumulation by cyclosporine. *J. Natl Cancer Inst.*, **82**, 1133.

HOLMES, J., WAREING, C., JACOBS, A., HAYES, J.D., PADUA, R.A. & WOLF, C.R. (1990). Glutathione-s-transferase pi expression in leukaemia: A comparative analysis with mdr-1 data. *Br. J. Cancer*, **62**, 209.

ITO, Y., TANIMOTO, M., KUMAZAWA, T. & 4 others (1989). Increased P-glycoprotein expression and multidrug-resistant gene (mdr1) amplification are infrequently found in fresh acute leukemia cells. *Cancer*, **63**, 1534.

JULIANO, R.L. & LING, V. (1976). A surface glycoprotein modulating drug permeability in chinese hamster ovary cell mutants. *Biochim. Biophys. Acta*, **455**, 152.

KARTNER, N., EVERNDEN-PORELLE, D., BRADLEY, G. & LING, V. (1985). Detection of P-glycoprotein in multidrug-resistant cell lines by monoclonal antibodies. *Nature*, **316**, 820.

KUWAZURU, Y., YOUHIMURA, A., HANADA, S. & 7 others (1990). Expression of the multidrug transporter, P-glycoprotein, in acute leukemia cells and correlation to clinical drug resistance. *Cancer*, **66**, 868.

LABARCA, C. & PAIGEN, K. (1980). A simple, rapid, and sensitive DNA assay procedure. *Anal. Biochem.*, **102**, 344.

MARIE, J.P., ZITTOUN, R. & SIKIC, B.I. (1991). Multidrug resistance (mdr1) gene expression in adult leukemias: correlations with treatment outcome and *in vitro* drug sensitivity. *Blood*, **78**, 586.

MUSTO, P., MELILLO, L., LOMBARDI, G., MATERA, R., DI GIORGIO, G. & CAROTENUTO, M. (1991). High risk of early resistant relapse for leukaemic patients with presence of multidrug resistance associated P-glycoprotein positive cells in complete remission. *Br. J. Haematol.*, **77**, 50.

NOONAN, K.E., BECK, C., HOLZMAYER, T.A. & 9 others (1990). Quantative analysis of MDR1 (multidrug resistance) gene in human tumors by polymerase chain reaction. *Proc. Natl Acad. Sci. USA*, **87**, 7160.

NYGREN, P., LARSSON, R., GRUBER, A., PETERSON, C. & BERGH, J. (1991). Doxorubicin selected multidrug-resistant small cell lung cancer cell lines characterised by elevated cytoplasmic $Ca^{2+}$ and resistance modulation by verapamil in absence of P-glycoprotein overexpression. *Br. J.Cancer*, **64**, 1011.

PETO, R., PIKE, M.C., ARMITAGE, P. & 7 others (1977). Design and analysis of randomized clinical trials requiring prolonged observation of each patient. II. Analysis and examples. *Br. J. Cancer*, **35**, 1.

PIRKER, R., WALLNER, J., GEISSLER, K. & 9 others (1991). MDR1 gene expression and treatment outcome in acute myeloid leukemia. *J. Natl Cancer Inst.*, **83**, 708.

SALMON, S.E., DALTON, W.S., GROGAN, T.M. & 4 others (1991). Multidrug-resistant myeloma: Laboratory and clinical effects of verapamil as a chemosensitizer. *Blood*, **78**, 44.

SATO, H., PREISLER, H., DAY, R. & 10 others (1990). MDR1 transcript levels as an indication of resistant disease in acute myelogenous leukaemia. *Br. J. Haematol.*, **75**, 340.

SCHUURHUIS, G.J., BROXTERMAN, H.J., DELANGE, J.H.M. & 9 others (1991). Early multidrug reistance, defined by changes in intracellular doxorubicin distribution, independent of P-glycoprotein. *Br. J. Cancer*, **64**, 857.

SONNEVELD, P. & NOOTER, K. (1990). Reversal of drug-resistance by cyclosporin-A in a patient with acute myelocytic leukemia. *Br. J. Haematol.*, **75**, 208.

STEEN, A.M., LUTHMAN, H., HELLGREN, D. & LAMBERT, B. (1990). Levels of hypoxanthine phosphoribosyltransferase RNA in human cells. *Exp. Cell Res.*, **186**, 236.

TAYLOR, C.W., DALTON, W.S., PARRISH, P.R. & 5 others (1991). Different mechanisms of decreased drug accumulation in doxorubicin and mitoxantrone resistant variants of the MCF7 human breast cancer cell line. *Br. J. Cancer*, **63**, 923.

THIEBAUT, F., TSURUO, T., HAMADA, H., GOTTESMAN, M.M., PASTAN, I. & WILLINGHAM, M.C. (1987). Cellular localization of the multidrug-resistance gene product P-glycoprotein in normal human tissues. *Proc. Natl Acad. Sci. USA*, **88**, 7735.

TSURUO, T., IIDA, H., NOJIRI, M., TSUKAGOSHI, S. & SAKURAI, Y. (1983). Circumvention of vincristine and adriamycin resistance *in vitro* and *in vivo* by calcium influx blockers. *Cancer Res.*, **43**, 2905.

TWENTYMAN, P.R., REEVE, J.G., KOCH, G. & WRIGHT, K.A. (1990). Chemosensitisation by verapamil and cylcosporin A in mouse tumor cells expressing different levels of P-glycoprotein and CP22 (sorcin). *Br. J. Cancer*, **62**, 89.

UEDA, K., CARDARELLI, C., GOTTESMAN, M.M. & PASTAN, I. (1987*a*). Expression of a full-length cDNA for the human 'MDR1' gene confers resistance to colchicine, doxorubicin, and vinblastine. *Proc. Natl Acad. Sci. USA*, **84**, 3004.

UEDA, K., CLARK, D.P., CHEN, C., RONINSON, I.B., GOTTESMAN, M.M. & PASTAN, I. (1987*b*). The human multidrug resistance (mdr1) gene. cDNA cloning and transcription initiation. *J. Biol. Chem.*, **262**, 505.

VAN DER BLIEK, A.M., BAAS, F., TEN HOUTE DE LANGE, T., KOOIMAN, P.M. VAN DER VELDE-KOERTS, T. & BORST, P. (1987). The human mdr3 gene encodes a novel P-glycoprotein homologue and gives rise to alternatively spliced mRNAs in liver. *EMBO J.*, **6**, 3325.

VAN DER BLIEK, A.M., BAAS, F., VAN DER VELDE-KOERTS, T. & 6 others (1988). Genes amplifed and overexpressed in human multidrug-resistant cell lines. *Cancer Res.*, **48**, 5927.